# The GIP Receptor Displays Higher Basal Activity than the GLP-1 Receptor but Does Not Recruit GRK2 or Arrestin3 Effectively

**DOI:** 10.1371/journal.pone.0106890

**Published:** 2014-09-05

**Authors:** Suleiman Al-Sabah, Munya Al-Fulaij, Ghina Shaaban, Hanadi A. Ahmed, Rosalind J. Mann, Dan Donnelly, Moritz Bünemann, Cornelius Krasel

**Affiliations:** 1 Department of Pharmacology and Toxicology, Faculty of Medicine, Kuwait University, Safat, Kuwait; 2 School of Biomedical Sciences, University of Leeds, Leeds, United Kingdom; 3 School of Pharmacy, Institute for Pharmacology and Toxicology, The Philipps University of Marburg, Marburg, Germany; University of Lancaster, United Kingdom

## Abstract

**Background and Objectives:**

Glucagon-like peptide-1 (GLP-1) and glucose-dependent insulinotropic polypeptide (GIP) are important regulators of insulin secretion, and their functional loss is an early characteristic of type 2 diabetes mellitus (T2DM). Pharmacological levels of GLP-1, but not GIP, can overcome this loss. GLP-1 and GIP exert their insulinotropic effects through their respective receptors expressed on pancreatic β-cells. Both the GLP-1 receptor (GLP-1R) and the GIP receptor (GIPR) are members of the secretin family of G protein-coupled receptors (GPCRs) and couple positively to adenylate cyclase. We compared the signalling properties of these two receptors to gain further insight into why GLP-1, but not GIP, remains insulinotropic in T2DM patients.

**Methods:**

GLP-1R and GIPR were transiently expressed in HEK-293 cells, and basal and ligand-induced cAMP production were investigated using a cAMP-responsive luciferase reporter gene assay. Arrestin3 (Arr3) recruitment to the two receptors was investigated using enzyme fragment complementation, confocal microscopy and fluorescence resonance energy transfer (FRET).

**Results:**

GIPR displayed significantly higher (*P*<0.05) ligand-independent activity than GLP-1R. Arr3 displayed a robust translocation to agonist-stimulated GLP-1R but not to GIPR. These observations were confirmed in FRET experiments, in which GLP-1 stimulated the recruitment of both GPCR kinase 2 (GRK2) and Arr3 to GLP-1R. These interactions were not reversed upon agonist washout. In contrast, GIP did not stimulate recruitment of either GRK2 or Arr3 to its receptor. Interestingly, arrestin remained at the plasma membrane even after prolonged (30 min) stimulation with GLP-1. Although the GLP-1R/arrestin interaction could not be reversed by agonist washout, GLP-1R and arrestin did not co-internalise, suggesting that GLP-1R is a class A receptor with regard to arrestin binding.

**Conclusions:**

GIPR displays higher basal activity than GLP-1R but does not effectively recruit GRK2 or Arr3.

## Introduction

Glucagon-like peptide-1 (GLP-1) and glucose-dependent insulinotropic polypeptide (GIP) are incretin hormones that function primarily to enhance glucose-stimulated insulin secretion [1], [2]. Their functional impairment is an early characteristic of type 2 diabetes mellitus (T2DM) [3]. Pharmacological levels of long-acting GLP-1 receptor (GLP-1R) agonists can overcome this impairment, and as a result, GLP-1R agonists are currently used clinically to treat T2DM [4]. In contrast, even at supra-physiological concentrations, GIP does not increase insulin secretion in patients with T2DM [5]. GLP-1R and GIPR are closely related members of the secretin family of G protein-coupled receptors (GPCRs) and positively couple to G proteins (Gα_s_), resulting in an increase in intracellular cyclic 3′-5′-cyclic adenosine monophosphate (cAMP) levels [6]. The actions of GLP-1 and GIP are not limited to pancreatic β-cells, and both peptides have numerous pleiotropic effects. For example, GLP-1 decreases appetite and may be cardio- and neuroprotective. GIP is involved in adipocyte metabolism and bone formation and may also have neuroprotective and neurotrophic effects [7]–[9]. Together, these actions make GLP-1R and GIPR exciting targets for the treatment not only of diabetes and obesity but also potentially of ischemic heart disease and neurodegenerative diseases, such as Alzheimer’s disease and Parkinson’s disease.

The canonical role of arrestins in regulating GPCR function is through homologous desensitisation and internalisation. Once a GPCR is activated, it becomes a substrate for GPCR kinases (GRKs), which phosphorylate specific serine and threonine residues in the receptor’s C-terminal tail and the 3^rd^ intracellular loop region. The GPCR can still signal through G proteins when phosphorylated; however, phosphorylation allows arrestins to bind to the activated receptor, preventing any further interaction with G proteins [10]. Arrestin can also act as a scaffolding molecule allowing the receptor to interact with components of endocytotic machinery, such as clathrin, thus mediating internalisation as well as signalling through G protein-independent pathways, such as mitogen-activated protein (MAP) kinases, Src tyrosine kinases and ubiquitin ligases [11]. Recently, arrestins have been shown to mediate GLP-1’s insulinotropic as well as proliferative effects on pancreatic β-cells [12], [13]. Although GLP-1R is known to interact with arrestin [14], homologous desensitisation and internalisation of GLP-1R appear to be arrestin-independent processes [12]. In contrast, very is little is known regarding GIPR’s interaction with arrestin.

An active receptor conformation can either be facilitated by agonist binding or can occur in constitutively active receptors (that is, receptors that preferentially adopt an active conformation in the absence of agonist). Several diseases are caused by mutations that result in constitutively active receptors (e.g., retinitis pigmentosa) [15]; however, constitutive activity can also be a property of certain wild-type receptors (e.g., the ghrelin receptor) [16].

In this study we sought to compare GLP-1R and GIPR’s basal activity and the ability of GLP-1 and GIP to stimulate GRK2 and arrestin3 (Arr3) recruitment to their respective receptors using a luciferase-based reporter gene assay, enzyme fragment complementation, confocal microscopy and single cell, real-time fluorescence resonance energy transfer (FRET).

## Materials and Methods

### Construction of cDNA

cDNA encoding human GLP-1R and GIPR subcloned into pcDNA1.1 were gifts from Martin Beinborn and Alan Kopin (Tufts University, USA). GLP-1R and GIPR were subsequently re-ligated into pcDNA3.1 (Invitrogen, Paisley, UK). cDNA encoding C-terminally YFP-labelled GLP-1R and GIPR (GLP-1-YFP and GIPR-YFP) were purchased from Source Bioscience (Nottingham, UK). GLP-1R and GIPR both possess a putative N-terminal signal peptide that is cleaved during receptor processing and trafficking [17], [18]. Therefore, to label the receptors at their N-termini, a myc-tag was introduced immediately downstream of the predicted signal peptide. To achieve this, pcDNA3 was modified by the addition of a linker region encoding the influenza hemagglutinin signal peptide (MKTIIALSYIFCLVFAA) between the KpnI and NotI sites of the multiple cloning site to produce pcDNA3-hgSP. The linker was constructed by annealing two complementary primers containing the hemagglutinin signal peptide sequence and KpnI and NotI restriction sites. A myc-tag (EQKLISEEDL) was introduced immediately downstream of the predicted signal peptide of GLP-1R and GIPR by sequential overlapping PCR using primers, which also added a NotI and XbaI site to the products’ termini. These products were then ligated into pcDNA3-hsSP to produce myc-GLP-1R and myc-GIPR. The constructs were verified through sequencing.

The cDNAs for YFP- and CFP-labelled arrestin 3 (Arr3-YFP, Arr3-CFP) have been previously described [19]. CFP-labelled GRK2 (GRK2-CFP) was constructed by amplifying the open reading frame of human GRK2 with suitable primers which added a HindIII site in front of the start codon and replaced the stop codon with an XbaI site. The resulting PCR product was cloned into Arr3-CFP (this construct used the enhanced version of CFP; mTurquoise) that had previously been cut with HindIII and XbaI to remove the Arr3 open reading frame. In essence, this strategy replaces the Arr3 open reading frame with that of GRK2. The construct was verified through sequencing.

### Ligands

Human GLP-1 (7–36) NH2 and human GIP (1–42) were purchased from Bachem (Bubendorf, Switzerland).

### Cell culture and transfection

Human embryonic kidney 293 (HEK-293) cells (ECACC Cat. no. 85120602) were cultured in Dulbecco’s modified Eagle’s media supplemented with 10% foetal calf serum, 100 U/ml penicillin and 100 µg/ml streptomycin. Chinese Hamster Ovary (CHO) cells stably expressing GLP-1R or GIPR and Arr3 were maintained in media provided by DiscoveRx (DiscoveRx Corporation Ltd., Birmingham, UK). Cells were maintained at 37°C in a humidified environment containing 5% CO_2_. HEK-293 cells were transiently transfected using Effectene (Qiagen, Hilden, Germany), following the manufacturer’s protocol.

### Luciferase assay

Activation of GLP-1R and GIPR was assessed by a luciferase reporter gene assay using the protocol described by Al-Fulaij *et al*. [20]. Briefly, HEK-293 cells were transiently transfected with cDNA encoding either GLP-1 or GIP receptor and a reporter gene construct consisting of a cAMP-response element fused to a reporter gene encoding firefly luciferase (Cre-luc) using Effectene (Qiagen, Hilden, Germany), following the manufacturer’s protocol. Twenty-four hours after transfection, the cells were seeded into white 96-well plates (Thermo Scientific, Roskilde, Denmark) at a density of 10,000 cells/well. Twenty-four hours later, the cells were incubated for 3 hours in media containing peptide ligand and then lysed. Luciferase activity was quantified using LucLite reagent (PerkinElmer Life and Analytic Sciences, Wellesley, MA, USA).

### Western blot analyses

Western blotting to detect myc-tagged GLP-1R and GIPR was performed as described by Akhtar *et al.* [21]. To compare the relative expression levels of mycGLP-1R and mycGIPR, HEK-293 cells were transiently transfected with equal amounts of cDNA encoding either mycGLP-1R or mycGIPR. Forty-eight hours after transfection, the cells were harvested and lysed in buffer (pH 7.6) containing 50 mM Tris-base, 5 mM EGTA, 150 mM NaCl, 1% Triton 100, 2 mM Na_3_VO_4_, 50 mM NAF, 1 mM PMSF, 20 µM phenylarsine, 10 mM sodium molybdate, 10 µg/ml leupeptin and 8 µg/ml aprotinin. Protein concentrations were estimated using the BioRad BCA protein assay. Samples containing equal amounts of protein were subjected to SDS-polyacrylamide gel electrophoresis (SDS-PAGE) and transferred onto nitrocellulose membranes (Schleicher & Schuell, Dassel, Germany). The membranes were then incubated with monoclonal antibodies produced in mouse to detect the myc-tagged receptors (Sigma, Germany Cat. no. M4439) followed by the secondary anti-mouse IgG horse-radish peroxidase-conjugated antibody (Sigma, Germany Cat. No. A9044). Immunoreactive bands were detected using SuperSignal chemiluminescent substrate (Pierce, UK) and Kodak autoradiography film (G.R.I., Rayne, U.K.). β-actin levels were detected using primary rabbit anti-human β-actin antibody (Sigma, Germany Cat. no. A2066) followed by the secondary goat anti-rabbit IgG horse-radish peroxidase-conjugated antibody (Santa Cruz Biotechnology, USA, Cat. no. SC 2030). Images were analysed and quantified by densitometry, and myc-immunoreactive bands were normalised to β-actin levels.

### Enzyme fragment complementation

Arrestin recruitment to GLP-1R or GIPR was investigated using the PathHunter eXpress kit (DiscoveRx). Briefly, the kit detects the interaction of arrestin with the activated receptor using enzyme fragment complementation. The β-galactosidase (β-gal) enzyme is split into two inactive fragments. The larger fragment (termed EA for enzyme acceptor) is fused to the C-terminal region of the arrestin molecule, and the smaller, 4-kDa fragment of β-gal (the ProLink) is fused to the receptors’ C-terminal tail. Upon receptor activation, the arrestin/receptor interaction brings the ProLink and EA fragments together, resulting in complementation of the two fragments of β-gal and the formation of a functional enzyme that hydrolyses the substrate and generates a chemiluminescent signal.

### Confocal and light Microscopy

HEK-293 cells transiently expressing Arr3-YFP and either GLP-1R or GIPR were plated on to poly-D-lysine-coated coverslips and mounted on to an “Attofluor” holder (Molecular Probes, Leiden, The Netherlands). The subcellular location of Arr3-YFP was monitored by live cell confocal microscopy performed on a Leica TCS SP5 system. YFP was excited with the 514 nm line of an argon laser, and images were captured using an oil-immersion 63× lens with the factory settings for YFP. Loss of cytoplasmic fluorescence over time was corrected for bleaching and quantified using the Leica confocal software. Assessment of the relative expression of GLP-1R-YFP and GIPR-YFP by comparing mean fluorescence intensity was performed in a similar manner.

### Fluorescence resonance energy transfer (FRET) measurements

HEK-293 cells were co-transfected with either GLP-1R-YFP or GIPR-YFP and either GRK2-CFP or Arr3-CFP. At 24 hours post-transfection, the cells were plated on poly-D-lysine-coated coverslips (25-mm diameter) in six-well plates. After 24 hours, FRET measurements were performed. Coverslips were mounted on a Nikon Eclipse TE2000S inverted microscope (Nikon, Kingston, UK) using an ‘Attofluor’ holder (Invitrogen, Leiden, The Netherlands), and the cells were superfused continuously with FRET buffer (137 mM NaCl, 5.4 mM KCl, 2 mM CaCl_2_, 1 mM MgCl_2_, 10 mM HEPES pH 7.3, 0.1% bovine serum albumin) using a computer-controlled rapid perfusion system (Ala-VC3-8SP, ALA Scientific Instruments). Ligands were dissolved in FRET buffer and applied using the same device. The cells were observed using an oil immersion 100x lens and excited using a CoolLED pE-2 (CoolLED, Andover, UK). Signals were detected using a EMCCD camera (Evolve512, Photometrics, Tucson, USA). The illumination time was set to 40 to 60 ms with a frequency of 5 Hz to minimise photobleaching. CFP was excited using 435 nm light, a 430/24 excitation filter and a 460 nm beamsplitter (Chroma Technology, Rockingham, VT), and fluorescence was measured at 535±15 nm (YFP) and 480±20 nm (CFP) through a beam splitter dichroic long-pass, 505 nm (Chroma Technology). The fluorescence signal at 535 nm is the sum of the YFP fluorescence and bleedthrough of CFP fluorescence into the YFP channel (approx. 40% of the fluorescence at 480 nm); therefore the “real” YFP fluorescence was calculated by subtracting the CFP bleedthrough from the F_535_ signal. FRET was calculated as F_YFP_/F_480_.

### Data analyses

Dose-response data were fitted to a sigmoidal curve, and kinetic experiments were fitted to mono-exponential decay curves using GraphPad 3.0 (GraphPad, San Diego, CA). The values are expressed as the mean ± standard error of the mean; n =  number of independent experiments. Statistical analysis of significance was calculated with GraphPad 3.0 using a two-tailed, unpaired Student’s *t*-test.

## Results

### Basal activity and activation of wild-type and modified GLP-1 and GIP receptors by their respective ligands and relative expression levels

**Figure 1:**
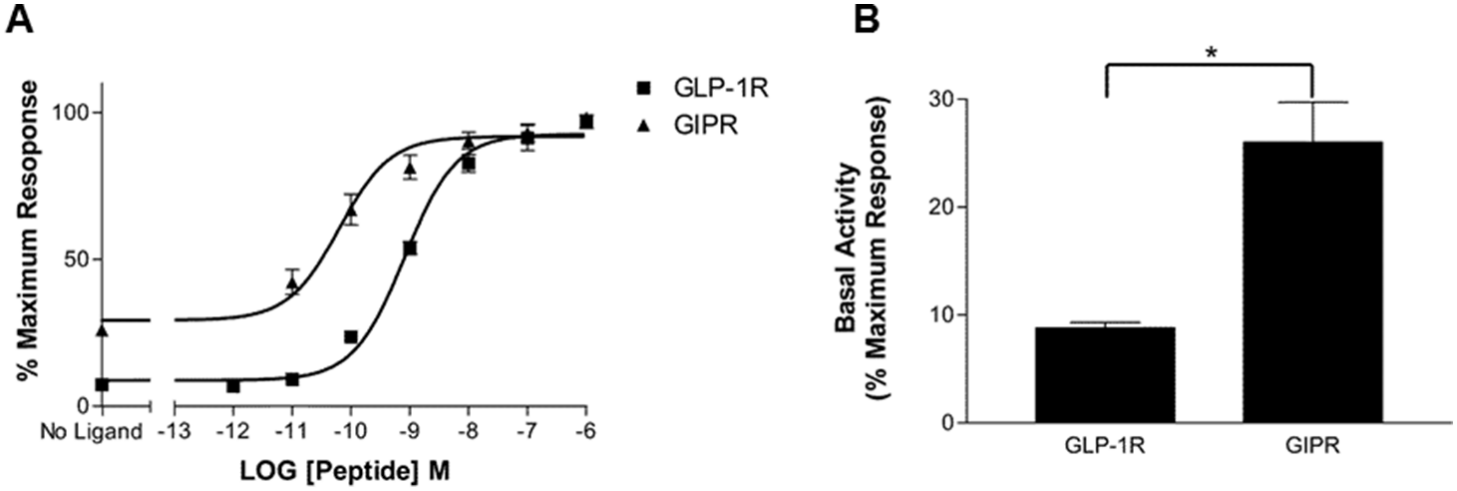
Luciferase assay and basal activity of GLP-1R and GIPR transiently transfected in HEK-293 cells. (A) GLP-1 and GIP stimulated cAMP-responsive luciferase activity in a dose-dependent manner in HEK-293 cells transiently expressing the corresponding receptor and reporter gene. (B) Basal activity of GLP-1R and GIPR expressed as a percentage of maximum stimulation. The results are expressed as the mean ± standard error of the mean for at least 3 independent experiments; *P<0.05.

**Figure 2:**
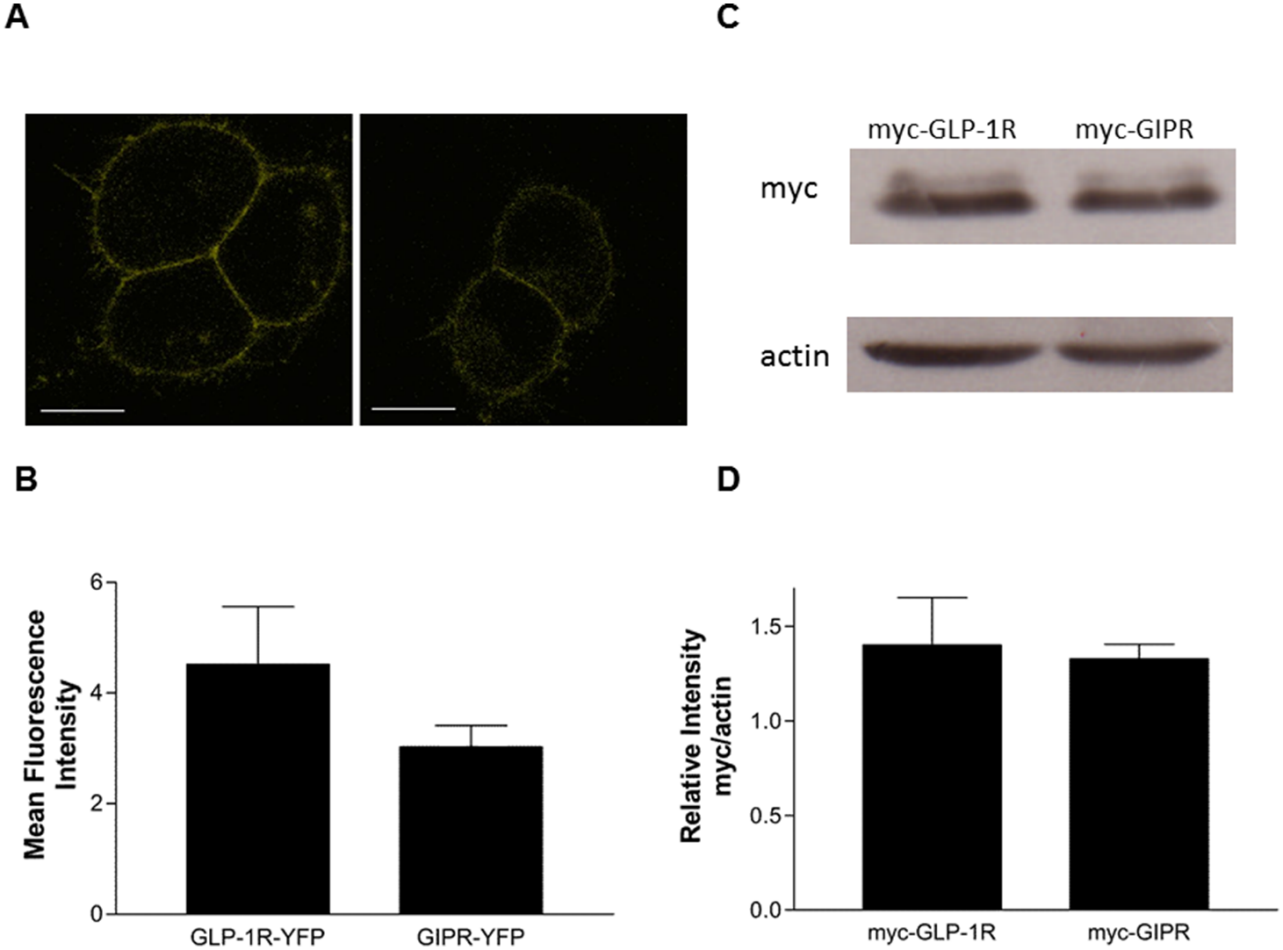
Relative expression of labelled GLP-1R and GIPR. (A) Representative confocal images of YFP-labelled receptors transiently expressed in HEK-293 cells; scale bar, 10 µm. (B) Mean fluorescence intensity measured from HEK-293 cells transiently expressing either GLP-1R-YFP or GIPR-YFP. (C) A representative Western blot showing protein levels of myc-labelled receptors and β-actin in HEK-293 cells transiently transfected with either myc-GLP-1R or myc-GIPR. (D) A densitometry histogram showing the relative expression levels of myc-GLP-1R and myc-GIPR normalised to actin. The results are expressed as the mean ± standard error of the mean for at least 5 independent experiments.

GLP-1 and GIP stimulated cAMP-responsive luciferase activity in a dose-dependent manner in HEK-293 cells transfected with Cre-luc and either wild-type GLP-1R or GIPR (Figure 1A, Table 1). Ligand-independent GIPR activity was significantly higher (*P*>0.05) than that of GLP-1R: 26% vs. 8.8%, respectively (Figure 1B, Table 1). As receptor expression levels can influence basal activity [22], the relative expression levels of GLP-1R and GIPR were assessed by comparing the mean fluorescence intensity of HEK-293 cells transiently expressing either GLP-1R-YFP or GIPR-YFP. The mean fluorescence intensity was slightly higher for GLP-1R-YFP than GIPR-YFP but did not reach significance (Figure 2B). Fusing YFP to the C-terminus of either GLP-1R or GIPR had no detectable effect on the potency of either ligand at its respective receptor, whereas GIPR-YFP’s basal activity was significantly lower than that of WT GIPR (Table 1). Nonetheless, GIPR-YFP’s basal activity was still significantly higher (*P*>0.05) than that of GLP-1R-YFP (Table 1). The relative expression of GLP-1R and GIPR was then assessed by comparing anti-myc immunoreactivity normalised to β-actin levels in HEK-293 cells transiently transfected with either myc-GLP-1R or myc-GIPR. There was no significant difference in the relative expression of either myc-GLP-1R or myc-GIPR (Figure 2D), and the introduction of the myc-tag to the N-terminus of GLP-1R and GIPR did not affect the potency of either ligand at its respective receptor nor the basal activity of either receptor (Table 1). At comparable levels of expression, myc-GIPR displayed significantly greater (*P*>0.001) ligand-independent activity than myc-GLP-1R (Figure 2D, Table 1).

**Table 1:** Basal activity and activation of WT and modified GLP-1 and GIP receptors by their respective ligands.

	*p*EC_50_	Basal Activity (% Maximum)
WT GLP-1R	9.1±0.04 (3)	8.8±0.5 (3)
GLP-1R-YFP	8.9±0.08 (3)	6.0±0.8 (3)
myc-GLP-1R	9.3±0.4 (3)	10.4±0.3 (3)
WT GIPR	10.2±0.3 (4)	*26.0±3.7 (4)
GIPR-YFP	10.0±0.1 (4)	* ^,^ #13.2±1.6 (4)
myc-GIPR	9.7±0.3 (4)	***31.1±1.0 (4)

The mean ± S.E.M shown are from at least 3 independent experiments (the number of experiments is shown in brackets). *p*EC_50_ refers to −logEC_50_/M.

*Basal activity significantly higher than corresponding GLP-1R modification (*P*<0.05),

# basal activity significantly lower than WT GIPR (*P*<0.05),

***basal activity significantly higher than the corresponding GLP-1R modification (*P*<0.001).

### Enzyme fragment complementation

**Figure 3:**
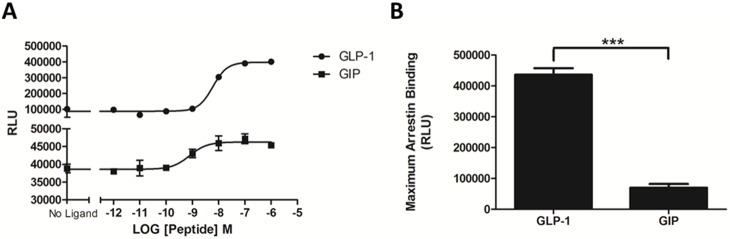
Enzyme fragment complementation (PathHunter) assay to monitor receptor/arrestin interaction. (A) GLP-1 and GIP stimulated the recruitment of arrestin to their respective receptors in a dose-dependent manner. Curves represent one of three independent experiments, where each data point represents the mean of triplicates, with S.E.M displayed as error bars. (B) Comparison of maximum arrestin binding. The results are expressed as the mean ± standard error of the mean for at least 3 independent experiments; ***indicates *P*<0.0001.

Using the PathHunter eXpress kit to measure arrestin-receptor interaction, GLP-1 and GIP stimulated the recruitment of arrestin to their cognate receptors in a dose-dependent manner, with *p*EC_50_ values of 8.2 (±0.14) and 8.1 (±0.27), respectively (mean ± S.E.M for 3 independent experiments; Figure 3A). However, maximum arrestin binding was 600% higher for GLP-1R than for GIPR (Figure 3B).

### Arrestin translocation to incretin receptors

**Figure 4:**
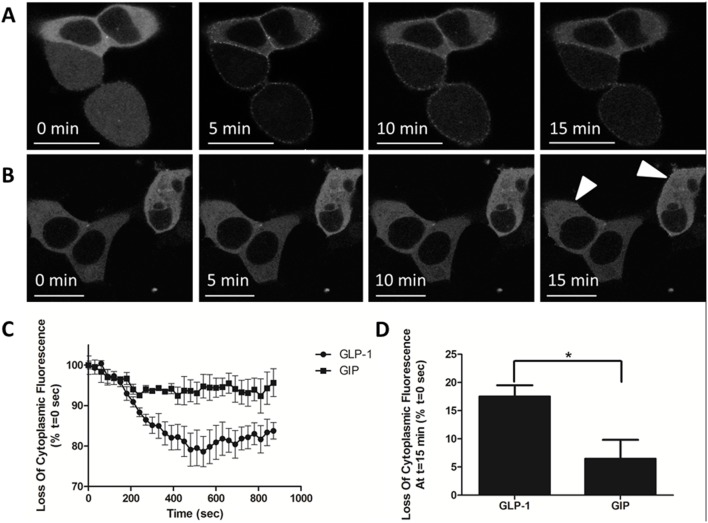
Agonist-stimulated arrestin translocation to the plasma membrane. HEK-293 cells were transiently transfected with Arr3-YFP and either (A) GLP-1R or (B) GIPR. Confocal images were captured every 30 s. A total of 1 µM of either (A) GLP-1 or (B) GIP was added immediately after the first image acquisition; scale bar, 20 µm. (C) Arrestin translocation was quantified as the loss of cytoplasmic fluorescence over time. (D) Loss of cytoplasmic fluorescence at 15 min; *indicates *P*<0.05. The results are expressed as the mean ± standard error of the mean for at least 4 independent experiments.

HEK-293 cells transiently co-transfected with Arr3-YFP and either GLP-1R or GIPR were plated on poly-D-lysine-coated coverslips and observed using a confocal microscope. Prior to agonist stimulation, arrestin was located in the cytosol in cells expressing GLP-1R or GIPR. After stimulating cells with 1×10^−6^ M agonist, arrestin translocation to the plasma membrane was apparent in the cells transfected with GLP-1R (Figure 4A) but only faintly detectable in the cells transfected with GIPR (Figure 4B). Arrestin translocation was quantified as the loss of cytoplasmic fluorescence over time (Figure 4C). At 15 min, the loss of cytoplasmic fluorescence stimulated by GLP-1 was significantly (*P*<0.05) greater than with GIP (Figure 4D).

### Single-cell FRET experiments show that GRK2 and Arrestin3 are recruited to agonist-stimulated GLP-1R but not GIPR

**Figure 5:**
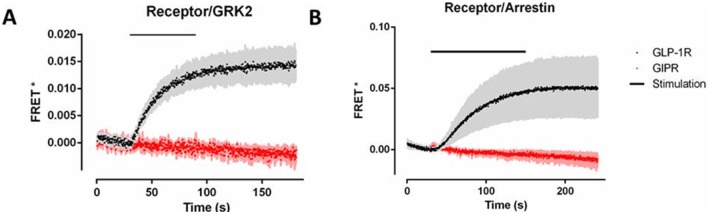
Agonist-induced FRET between receptor and (A) GRK2 and (B) Arrestin3. HEK-293 cells were transiently transfected with GLP-1R and GIPR and (A) GRK2-CFP (B) Arr3-CFP. The traces are the mean ± standard error of the mean for at least 5 independent experiments.

HEK-293 cells were transiently transfected with either GLP-1R-YFP (black traces) or GIPR-YFP (red traces) and either GRK2-CFP or Arr3-CFP. Upon agonist stimulation, GRK2 was recruited to GLP-1R with a rate constant of k = 0.040 s^−1^ (±0.005 SEM, n = 5) and a half-life of 17.2 s. Recruitment to agonist-stimulated GIPR was not detectable in the single-cell FRET assay (Figure 5A). In agreement with our previous experiments, agonist stimulation of GLP-1R resulted in robust arrestin recruitment with a rate of k = 0.017 s^−1^ (±0.002 SEM, n = 6) and a half-life of 39.7 s. Again, agonist stimulation of GIPR did not result in arrestin recruitment (Figure 5B). Both GRK2 and Arr3 remained bound to GLP-1R even after agonist washout.

### Light microscopy shows Arr3 remains at the membrane after prolonged GLP-1R stimulation

**Figure 6:**
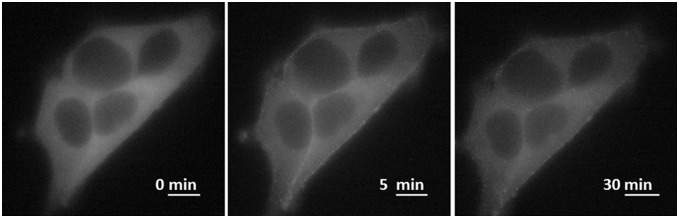
Light microscopy (37°C) shows that CFP-labelled arrestin3 remains at the plasma membrane 30 min after stimulation with GLP-1. Representative image of HEK-293 cells transiently transfected with GLP-1R and Arr3-CFP. A total of 1 µM of GLP-1 was added immediately after acquisition of the first image; scale bar, 10 µm.

HEK-293 cells were transiently transfected with Arr3-CFP and either GLP-1R or GIPR. Cells were observed under a light microscope at 37°C and stimulated with agonist. GLP-1R stimulation resulted in arrestin translocation to the plasma membrane. Arrestin remained at the plasma membrane 30 minutes after stimulation and did not co-internalise with GLP-1R (Figure 6).

## Discussion

GLP-1 and GIP are important regulators of glucose homeostasis and pancreatic β-cell function, and impairment of their effect is an early characteristic of T2DM. GLP-1R agonists are used clinically as anti-diabetic drugs, as are DPP-IV inhibitors, which prolong the circulating half-life of both endogenous GLP-1 and GIP [23]. To date, GIPR agonists are not used clinically; however, GLP-1R/GIPR co-agonists have recently been reported to have similar efficacy to GLP-1R agonists in terms of glucose control and superior efficacy in terms of weight loss [24]. Hence, a detailed understanding of the signalling mechanisms of the two incretin hormones is of great importance.

Using a luciferase-based reporter gene assay, we detected significantly higher constitutive activity in GIPR compared with GLP-1R (Figure 1A and B). Increased levels of receptor expression can amplify basal activity [22]; therefore, the relative levels of GLP-1R and GIPR expression were assessed. Both receptors were tagged with YFP at their C-termini and a myc-tag at the N-termini. No significant differences in expression levels were found using either mean fluorescence intensity (YFP-tagged) or Western blotting (myc-tagged; Figure 2). Neither modification affected the potency of GLP-1 or GIP at their respective receptor. However, the addition of YFP to GIPR’s C-terminus significantly reduced the receptor’s basal activity compared with WT GIPR, although GIPR-YFP still displayed significantly higher basal activity GLP-1R-YFP (Table 1). The addition of a myc-tag to the N-terminus of GLP-1R and GIPR did not significantly affect the basal activity of either receptor. Taken together, these data demonstrate that at comparable expression levels, GIPR displays significantly higher levels of ligand-independent activity than GLP-1R, which in contrast, is relatively silent. This finding is in agreement with previous work that also demonstrated that GIPR has a considerable degree of basal activity [25]; however, these studies did not compare this activity to that of GLP-1R when expressed at similar levels. It should be noted that, based on quantitative RT-PCR, GLP-1R is expressed at 10 times the level of GIPR in pancreatic islets [26]. Nonetheless, a glutamic acid to glutamine substitution at position 354 in GIPR’s 6^th^ transmembrane domain results in lowered basal activity. Subjects homozygous for the E354Q polymorphism were found to have reduced fasting and post oral glucose tolerance test serum C-peptide concentration (an indicator of insulin secretion) [27], suggesting that GIPR’s constitutive activity may play a role in glucose homeostasis. More recently, the same GIPR polymorphism has been shown to be associated with reduced bone mineral density and increased fracture risk, suggesting a role for GIPR’s basal activity in osteoblast function [28].

The traditional role for GRKs and arrestins is to mediate the homologous desensitisation and internalisation of GPCRs, as well as activation of tyrosine kinase signalling pathways. Arr2 has been shown to mediate GLP-1 signalling in cultured pancreatic β-cells. Knockdown of Arr2 by RNAi reduced GLP-1-stimulated cAMP levels and impaired GLP-1-stimulated insulin secretion [12]. Interestingly, Arr2 knockdown did not affect GLP-1R desensitisation or internalisation. Arr3-knockout mice displayed impaired glucose tolerance and insulin secretion; however, GLP-1 amplification of insulin secretion was not affected [29]. In contrast, very little is known regarding the interaction between GIPR and either GRKs or arrestins. We hypothesised that given GIPR’s high level of basal activity observed in the luciferase assay, GIPR may interact with arrestin in a ligand-independent manner.

Several methods were employed to compare the ability of GLP-1R and GIPR to interact with Arr3. Initially, we used a commercially available enzyme fragment complementation assay (PathHunter, DiscoveRx) to investigate arrestin recruitment to GLP1-R and GIPR. GLP-1 and GIP stimulated Arr3 recruitment to their respective receptors with comparable potency (Figure 3A). However, the signal window for GIPR was substantially smaller than for GLP-1R, and as a result, maximum arrestin binding was also significantly lower (Figure 3B). Due to the nature of this assay, we were unable to compare or manipulate variables such as the relative expression of arrestin and/or receptors; therefore, alternative methods were employed.

Translocation of YFP-labelled Arr3 to agonist-stimulated GLP-1R and GIPR expressed in HEK-293 cells was monitored using confocal microscopy. We found that GLP-1 stimulated a robust translocation of arrestin to the plasma membrane (Figure 4A), which is in agreement with the work of Jorgensen *et al.* [30], who used bioluminescence resonance energy transfer (BRET) to investigate GLP-1R/Arr3 interactions, and in contradiction to the work of Syme *et al*, who used essentially the same experimental design as we did but could not demonstrate recruitment of Arr3 to agonist-stimulated GLP-1R [31]. Others have also shown the GLP-1R interacts with both Arr2 and Arr3 [30], [32]. However, GLP-1R endocytosis appears to be an arrestin-independent process, which is in agreement with Syme *et al*., who reported a role for caveolin-1 in this process. In contrast to GLP-1R, arrestin recruitment to agonist-stimulated GIPR was only faintly detectable after 15 minutes of stimulation (Figure 2B). There is a lack of information in the literature regarding the interaction between GIPR and arrestins. Co-expression of Arr2 with GIPR impaired GIP-mediated cAMP production and insulin release in HEK-293 cells and betaTC3 cells, respectively, but no direct interaction between GIPR and Arr2 has been demonstrated [33]. Recent studies using BRET have shown that GLP-1 can induce heterodimerisation of GLP-1R and GIPR, whereas treatment with GIP reversed dimer formation. Intriguingly, co-expression of GLP-1R and GIPR reduced GLP-1-stimulated arrestin recruitment to GLP-1R. This result suggests that GIPR can act as a negative regulator of arrestin binding to GLP-1R and is consistent with our data that show that GIPR poorly recruits arrestin [32].

Using single cell FRET measurements, we investigated the kinetics of GRK2 and Arr3 recruitment to agonist-stimulated GLP-1R and GIPR (Figure 5A and B). GLP-1 stimulated GRK2 recruitment to GLP-1R with faster kinetics than Arr3 (k = 0.040 s^−1^ and k = 0.017 s^−1^, respectively). Previously, studies using BRET to investigate the kinetics of GRK2 and Arr3 interaction with GLP-1R produced similar results, with a faster time course being observed for GRK2 than Arr3 [34]. The authors propose a model whereby Arr3 competes with GRK2 for interaction with the phosphorylated receptor. Although our data are consistent with this model, we observed that Arr3 was recruited to GLP-1R in one phase as opposed to two phases as observed by Jorgensen *et al*. It is possible that this difference is due to the different assays used to monitor the time course of GLP-1R/Arr3 interaction. Single-cell FRET allows for greater temporal resolution than BRET, which also measures interactions in cell populations as opposed to single cells. The two phases are explained as phosphorylation-independent and -dependent arrestin recruitment. We did not detect an initial phosphorylation-independent phase for Arr3 recruitment to GLP-1R. A two-phase arrestin association has previously been observed for the β2-adrenergic receptor, and an alternative explanation is that the first phase is due to arrestin recruitment to pre-phosphorylated receptors [10]. The time course in our experiments was comparable to arrestin recruitment to the parathyroid receptor, which also displays a one-phase association [35]. Again, in contrast to GLP-1R and in agreement with our previous experiments, GIPR stimulation did not result in either GRK2 or arrestin recruitment. GRK2 overexpression has been shown to increase agonist-mediated GIPR phosphorylation; however, this has not been demonstrated to be a direct effect [33]. To our knowledge, a direct interaction between GIPR and GRK2 has only been demonstrated through immunoprecipitation assays in adipocytes [36]. This difference may be due to the cell type or the method used to assess GIPR/GRK2 interaction. It is also possible that the addition of the YFP molecule to the C-terminus of GIPR prevents the receptor from interacting with GRK2 or Arr3. This possibility is unlikely, however, as YFP-labelled GLP-1R was able to interact with both GRK2 and Arr3, and the receptors share similar sequences.

GPCRs can be classified by their interactions with arrestin. Class A receptors interact with arrestin transiently at the plasma membrane after agonist stimulation, whereas class B receptors co-internalise with arrestin [37]. Although GLP-1R and Arr3 remained associated after agonist washout in the FRET assay, a characteristic of Class B receptors, our experiments using light microscopy showed that Arr3 remained at the plasma membrane even after prolonged stimulation of GLP-1R at 37°C (Figure 4). These data suggest that GLP1-R is a class A receptor in terms of arrestin binding. The failure of agonist washout to dissociate GLP-1R from Arr3 is likely to relate to the off-rate of GLP-1 from GLP-1R.

In conclusion, we demonstrate that at comparable levels of expression, GIPR has significantly higher levels of basal activity than GLP-1R. Furthermore, whereas agonist stimulation of GLP-1R results in robust recruitment of GRK2 and Arr3, the same is not true for GIPR. We also demonstrate that GLP-1R behaves like a class A receptor in terms of arrestin binding. As these two receptors share considerable sequence homology, especially in their C-terminal regions, future experiments should investigate the molecular determinants for this differential recruitment of GRK2 and Arr3. The interaction between GLP-1R and GIPR and other members of the GRK and arrestin family should also be investigated.
